# Spectrum of Opportunistic Fungal Infections in HIV/AIDS Patients in Tertiary Care Hospital in India

**DOI:** 10.1155/2016/2373424

**Published:** 2016-06-20

**Authors:** Ravinder Kaur, Megh S. Dhakad, Ritu Goyal, Preena Bhalla, Richa Dewan

**Affiliations:** ^1^Department of Microbiology, Lady Hardinge Medical College and Associated Hospitals, New Delhi 110001, India; ^2^Department of Microbiology, Maulana Azad Medical College and Associated Lok Nayak Hospitals, New Delhi 110002, India; ^3^Department of Medicine, Maulana Azad Medical College and Associated Lok Nayak Hospitals, New Delhi 110002, India

## Abstract

HIV related opportunistic fungal infections (OFIs) continue to cause morbidity and mortality in HIV infected patients. The objective for this prospective study is to elucidate the prevalence and spectrum of common OFIs in HIV/AIDS patients in north India. Relevant clinical samples were collected from symptomatic HIV positive patients (*n* = 280) of all age groups and both sexes and subjected to direct microscopy and fungal culture. Identification as well as speciation of the fungal isolates was done as per the standard recommended methods. CD4+T cell counts were determined by flow cytometry using Fluorescent Activated Cell Sorter Count system. 215 fungal isolates were isolated with the isolation rate of 41.1%.* Candida* species (86.5%) were the commonest followed by* Aspergillus* (6.5%),* Cryptococcus* (3.3%),* Penicillium* (1.9%), and* Alternaria* and* Rhodotorula* spp. (0.9% each). Among* Candida* species,* Candida albicans* (75.8%) was the most prevalent species followed by* C. tropicalis* (9.7%),* C. krusei* (6.4%),* C. glabrata* (4.3%),* C. parapsilosis* (2.7%), and* C. kefyr* (1.1%). Study demonstrates that the oropharyngeal candidiasis is the commonest among different OFIs and would help to increase the awareness of clinicians in diagnosis and early treatment of these infections helping in the proper management of the patients especially in resource limited countries like ours.

## 1. Introduction

The clinical profile of AIDS in India is seen to be different from what is seen in the developed world, since the HIV infected individual in India lives in an environment with high prevalence of infectious diseases [1]. The major causes of morbidity and mortality in HIV infected patients are the opportunistic infections (OIs). This could be attributed to the decreased level of immunity in such patients due to destruction of CD4+ cells. Thus, these patients become vulnerable to various OIs, particularly those caused by fungi [2, 3].

There are major differences in the spectrum of OIs in India and in the west [1, 4]. Among the OFIs,* Candida albicans*,* Cryptococcus neoformans*, and* Aspergillus fumigatus* infections have accounted for most of the mycotic infections in immunocompromised individuals, and these infections often become life threatening [5–8]. Although* Candida albicans* have been found to be the most commonly isolated organism, some studies have shown that non-albicans* Candida *species including* C. tropicalis*,* C. krusei*, and* C. glabrata* are more prevalent than* C. albicans* [3].

The incidence of OFIs has increased, especially in hospitalised patients with the greatest risk [8] as in HIV infected patients. An early specific diagnosis and subsequent treatment to combat these infections are not only the concern of western hospitals but also equally relevant to developing countries like ours. In India, diagnosis as well as surveillance of these OFIs in AIDS is not easy, as there are not many laboratory setups presently to be able to deal with specific diagnosis of infection. In order to get an insight into the present scenario of the patients with HIV infection and AIDS in an Indian setup, the present study was planned to understand the prevalence and spectrum of common OFIs in HIV/AIDS patients.

## 2. Material and Methods

This study was approved by the institutional ethics committee, Maulana Azad Medical College and Associated Hospitals (Lok Nayak, GB Pant Hospital, Guru Nanak Eye Centre, and Chacha Nehru Bal Chikitsalaya), New Delhi, India. Individual informed consent was obtained from the patients.

### 2.1. Study Population and Design

**Figure 1:**
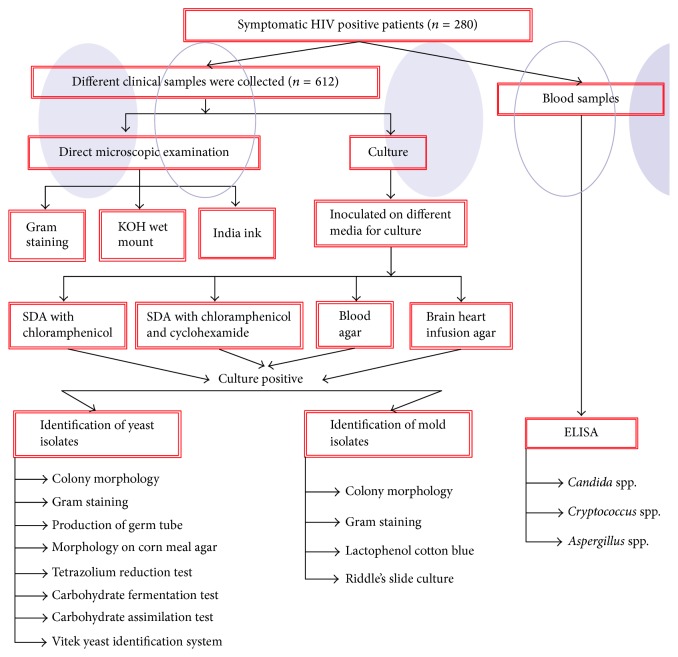
Flowchart of the study.

Two hundred eighty patients (*n* = 280) of all age groups and both sexes attending outpatient departments (OPDs) or antiretroviral treatment clinic (ART clinic) or admitted in the medical wards of LNJP were studied. All patients were evaluated by a predesigned protocol covering the biodata, history including mode of transmission, presenting complaints, and physical examination.  Figure 1 shows the flowchart of the study.

### 2.2. Microscopy, Culture, and Identification

Depending on the clinical symptoms and organ system involved, relevant clinical samples were collected with complete universal precautions. The samples were subjected to direct microscopy using Gram staining, KOH mounts, and India ink preparations, depending on the type of specimen and the suspected infection in the patient. Standard recommended procedures were used for diagnosis and isolation, which included a battery of tests [14, 15].

Fungal culture was done on Sabouraud dextrose agar with chloramphenicol (16 mg/mL) and with and without cycloheximide, blood agar, and brain heart infusion agar. Specimens were streaked in duplicate; one set of inoculated slants was incubated at 25°C and the other at 37°C, and they were examined every other day for growth up to 4–6 weeks before discarding as negative. Samples inoculated on blood agar were incubated for 24–48 h and samples on brain heart infusion agar were incubated for 1-2 weeks [14, 16]. Fungal growth was identified by colony morphology, Gram staining, lactophenol cotton blue preparation, and Riddle's slide culture as per standard recommended procedures [17]. Identification and speciation of yeast isolates were done on the basis of germ tube production, morphology on corn meal agar with Tween 80 (Hi Media), HiCrome candida agar (Hi Media), carbohydrate fermentation tests, assimilation tests using yeast nitrogen base agar (Hi Media) [14, 16, 17], and an automated Vitek-2 compact system (Biomérieux, India) as per standard recommended procedures.

### 2.3. Assessment of Immune Status

CD4 count was determined for each patient enrolled in our study by flow cytometry using the fluorescent activated cell sorter BD FACS Count system (Becton Dickinson) as per the manufacturer's instructions.

## 3. Results

Patients belonged to a wide age group (4–68 years). Maximum number of OFI cases was observed in 21–40 years (77%), the most productive age group of the country. We found that males (67.14%) were more commonly infected, and a male predominance was seen in most of the age groups (Table 1).

Heterosexual mode of transmission was the commonest (71%) route of HIV transmission. Clinically, patients presented with more than one symptom: the most common in our study population was weight loss (78%), followed by oral ulcers (75%), fever (67%), headache (54%), loss of appetite (44%), cough (40%), diarrhea (28%), dyspnoea (27%), neck rigidity (26%), and others (4–19%) (Table 2). AIDS defining illness like Tuberculosis was seen in 49.6% of patients followed by recurrent diarrhea (28%) and recurrent herpes zoster (1.3%). The gastrointestinal system was involved in all the patients, followed by the respiratory system (Table 2). Abdominal ultrasonographic study showed hepatosplenomegaly in 3.2% of patients and chest X-ray showed bilateral infiltrates in 5.4% of the patients.

**Figure 2:**
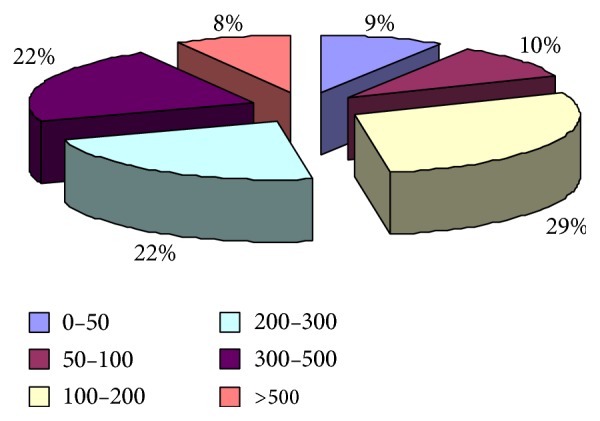
CD4 count profile of HIV/AIDS patients.

The CD4 count ranged from 0 to 500 in 92% patients while only 8% had a count of >500. 133 (47.5%) patients had CD4 counts <200 cells/*μ*L, while CD4 count <100 cells/*μ*L was seen in 52 (18.6%) and CD4 count <50 cells/*μ*L in 25 (8.9%) patients depicting a major population with severe immunosuppression (Figure 2).

A total of 612 samples were collected and processed from 280 patients. Detailed distribution of different fungal isolates among various clinical samples is shown in Table 3. A total of 215 fungal isolates were isolated,* Candida* spp. (86.5%) being the commonest followed by* Aspergillus *spp. (6.5%),* Cryptococcus* spp. (3.3%),* Penicillium* spp. (1.9%), and* Alternaria* spp. and* Rhodotorula* spp. (0.9% each).

Among the* Candida *isolates,* C. albicans* (141) was the most prevalent species followed by* C. tropicalis* (18),* C. krusei* (12),* C. glabrata* (8),* C. parapsilosis* (5), and* C. kefyr* (2). Among* Aspergillus* spp.,* A. niger* (7) was the most common followed by* A. fumigatus *(5) and* A. flavus* (2). Among* Cryptococcus *spp.,* C. neoformans* (5) was the most common followed by* C. gattii *(2). However, 4 of* Penicillium marneffei* and 2 each of* Alternaria alternata* and* Rhodotorula mucilaginosa* were isolated (Table 4). Final diagnosis of infectious complications showed fungemia in 55 (19.6%) cases followed by invasive candidiasis in 23 (8.2%), invasive aspergillosis in 12 (4.3%), and hepatosplenic candidosis in 9 (3.2%) cases.

## 4. Discussion

HIV related OFIs are an important cause of morbidity and mortality in the developing nations like ours. There are many reports available regarding the pattern of OIs in HIV infected individuals [18], but the data from India on the etiology and spectrum of fungal infections of these patients are scarce [19]. Our study divulges the spectrum of common OFIs in HIV/AIDS patients in a tertiary care hospital in north India.

In this study, majority of the patients belonged to 21–40 years (77%), the most productive age group of the country, showing a male preponderance with a male to female ratio of 2.25 : 1 consistent with studies on HIV patients in India and Iran [20, 21]. Preponderance of males may be due to their migration to the metropolitan cities in search of work. Staying away from their spouse for longer periods and the philandering habit of males being seen might have resulted in their acquiring HIV infection. Moreover, the male preponderance seen might have been due to the fact that in the existing social milieu in India females do not seek medical care because of fearing ostracism and loss of family support [22, 23].

According to Joshi et al. (2004) [24] study, weight loss (58.8%) was the most common clinical complaint, almost similar to our study. However, Gorantla et al. (2015) [25] observed fatigue and malaise (23%) followed by fever (16.8%), cough and dyspnoea (15.9%), diarrhea (10.2%), and weight loss (9.3%) to be the major symptoms among the HIV seropositive patients.

In our study,* Candida* species (86.5%) were the commonest followed by* Aspergillus* spp. (6.5%) and* Cryptococcus* spp. (3.3%) which is similar to the various studies as in Table 5 [9–13].

Among* Candida* species isolates,* C. albicans* (75.8%) was the most prevalent species followed by* C. tropicalis* (9.7%) almost similar to Gandham et al. (2013) [13] study from western part of India (2010 to 2012). However, in another study by Picardi et al. (2012) [26] in USA between 2004 and 2009 reported* Candida non-albicans* strains were more frequently isolated in neutropenic patients. Among* Aspergillus* spp.,* A. niger* (50%) was the most common followed by* A. fumigatus *(35.7%) and* A. flavus* (14.3%). However, Gandham et al. (2013) [13] reported* A. fumigatus *(53.2%) to be the most common followed by* A. niger* (25.5%) and* A. flavus* (14.9%). Among* Cryptococcus *spp.,* C. neoformans *(71.4%) was followed by* C. glutei *(28.6%) while in Gandham et al. (2013) [13] study only* C. neoformans* sp. (100%) was isolated from the immunocompromised patients.

The findings of Xiao et al. (2013) [27] revealed the prevalence of* Penicillium marneffei* (1.4%) to be almost similar to our findings (1.9%). Penicilliosis is an important HIV-associated opportunistic infection known to be endemic in Southeast Asia [28]. In our study* Alternaria alternata* (0.9%) were isolated in low frequency; a higher frequency (5.6%) has been isolated in a study on cancer/HIV patients from central India [29]. All the isolates of* Alternaria alternata* were isolated from the respiratory samples of the patients,* Alternaria alternata* being thought to be the main airborne allergen of the genus Alternaria [30].

*Rhodotorula* species are generally considered to be nonpathogenic and have rarely been a cause of human infection [31]. In our study* Rhodotorula *species were isolated in low frequency, that is, 0.9%, similar to a study from Brazil in 2010 [8]. However, ARTEMIS surveillance project reported that* Rhodotorula* species were the fourth most prevalent noncandidal yeast (4.2%) isolated from clinical specimens [5].

In our study,* Candida* species was the most common isolate in oropharyngeal samples (49.3%). Oropharyngeal candidiasis is the most common opportunistic fungal infection reported in many studies [3, 32–34]. The occurrence of oral candidiasis is recognized as an indicator of immune suppression and is often found in HIV infected patients with CD4 counts fewer than 200 cells/*μ*L [27].

In sputum also* Candida* spp. (32.9%) predominate followed by* Aspergillus *spp. (13.2%); the relative proportions seen in this study reflect those seen by Bharathi and Rani, but absolute values are very different [35]. However, in blood* Candida* spp. are the fourth most common pathogen isolated from the blood of hospitalized patients [36]. In our study in blood, the prevalence of* Candida* infection (8.9%) was higher, although a lot of variation in the prevalence and incidence of candidemia has been reported in different places in India varying from 1.6% to 6.9% [36–39].

In urine samples,* Candida* spp. (15.1%) and* Aspergillus *spp. (6.1%) were isolated in our study. A point prevalence survey done in 228 hospitals from 29 European countries determined that 9.4% of nosocomial UTIs were caused by* Candida* spp. Depending on the population examined,* Candida* is reported in up to 44% of urine samples sent for culture. Two retrospective analyses done in Israel and Italy found much lower rates (varying between 0 and 1.4%) in urine cultures [40].

In stool samples,* Candida* spp. (7.6%) and* Rhodotorula* spp. (5.1%) were isolated in our study. However, Anwar Khan et al. (2012) [41] reported 5% of patients had* Candida* diarrhea while Magalhães et al. (2015) [8] reported 0.9%* Candida* spp. isolation from stool samples of the hospitalized patients in Brazil in 2010.* Rhodotorula* spp. have been isolated from stool samples, indicating that these yeasts can survive in the extreme conditions of the gastrointestinal tract with recent studies having demonstrated an incidence of fungemia caused by* Rhodotorula *between 0.5% and 2.3% in the USA and Europe, respectively [42].

In CNS infections in our study, Cryptococcosis (8.2%) was the most prevalent opportunistic fungal infection. However, Jain et al. [3], Chakraborty et al. [43], Sharma et al. [44], and Mulla et al. [34] reported a low incidence of Cryptococcosis (6.7%, 4%, 3.7%, and 2.9%, resp.). Cryptococcosis is the most common systemic fungal infection among AIDS patients and its incidence is on the rise with the rapid spread of the disease [3, 45]. CNS Cryptococcosis is one of the most important risk factors associated with HIV infection contributing to a very high degree of morbidity and mortality among HIV infected patients [3, 46].

For the prevention of OFIs, specific safety measures should be adopted such as good personal hygiene, early and regular medical examination, prompt diagnosis, and appropriate antifungal prophylaxis/treatment. These are necessary to decrease the morbidity and mortality associated with these infections in HIV infected patients, which in turn may increase their longevity [3].

Antifungal susceptibility testing plays an important role in managing and guiding therapeutic decision making especially for difficult to treat invasive candidiasis and aspergillosis. It also aids in drug development studies and as a means of tracking the development of antifungal resistance in epidemiological studies [45, 46].

In conclusion, oropharyngeal candidiasis was found to be most common OFIs with different fungal infections. This study would help to increase the awareness for clinicians to come up with right diagnosis and earlier treatment of these infections with the proper management of the patients especially in resource limited regions in India.

## Figures and Tables

**Table 1:** Age and sex distribution of the patients (*n* = 280).

Age group (in years)	Male		Female		Intersex		Total	
	*n*	%	*n*	%	*n*	%	*n*	%
0–10	1	0.35	0	0	0	0	1	0.35
11–20	5	1.79	2	0.72	2	0.71	9	3.22
21–30	66	23.58	43	15.35	0	0	109	38.93
31–40	74	26.42	28	10.00	5	1.79	107	38.21
41–50	32	11.42	9	3.22	0	0	41	14.64
51–60	7	2.5	2	0.72	0	0	9	3.22
61–70	3	1.08	1	0.35	0	0	4	1.43

Total	188	67.14	85	30.36	7	2.5	280	100

**Table 2:** Frequency of clinical presentations and system involvement in patients.

	Patient, *n*	Patient, %
*Clinical presentations*		
Weight loss	219	78.2
Oral ulcer	209	74.6
Fever	188	67.1
Headache	150	53.6
Loss of appetite	123	43.9
Cough	111	39.6
Diarrhea	79	28.2
Dyspnea	75	26.8
Neck rigidity	74	26.4
Other (skin rash, night sweats, painful swallowing, loss of memory, lymphadenopathy, sensory loss, burning micturition, vision loss)	299	4-19

*System involvement*		
GI	280	100
Respiratory	91	32.5
Cardiovascular	89	31.8
CNS	73	26.1
Colorectal	39	13.9
Genitourinary	36	12.9
Skin	4	1.4

**Table 3:** Distribution of different fungal isolates among various clinical samples.

Clinical samples (*n* = 612)	Patients		Fungal isolate distribution in clinical samples		
	Patient, *n*	Patient, %		Organism, *n*	Organism, %
Oropharyngeal	280	100	*Candida *spp.	138	49.3

Induced sputum	91	32.5	*Candida *spp.	30	33.0
			*Aspergillus *spp.	12	13.2
			*Penicillium *spp.	4	4.4
			*Alternaria *spp.	2	2.2
			*Cryptococcus *spp.	1	1.1

Blood	89	31.8	*Candida *spp.	8	9.0

CSF	73	26.2	*Cryptococcus *spp.	6	8.2
			*Candida *spp.	1	1.4

Stool	39	13.9	*Candida *spp.	3	7.7
			*Rhodotorula *spp.	2	5.1

Urine	33	11.8	*Candida *spp.	5	15.2
			*Aspergillus *spp.	2	6.1

Skin	4	1.4	No growth	—	—

Genital sample	3	1.1	*Candida *spp.	1	33.3

**Table 4:** Species distribution of different fungal isolates.

Fungal isolates (*n* = 215)	*n*	% of total
*Candida *spp.	186	86.5
*C. albicans*	141	75.8
*C. tropicalis*	18	9.7
*C. krusei*	12	6.5
*C. glabrata*	8	4.3
*C. parapsilosis*	5	2.7
*C. kefyr*	2	1.1

*Aspergillus *spp.	14	6.5
*A. niger*	7	50.0
*A. fumigatus*	5	35.7
*A. flavus*	2	14.3

*Cryptococcus *spp.	7	3.3
*C. neoformans*	5	71.4
*C. gattii*	2	28.6

*Penicillium marneffei*	4	1.9

*Alternaria alternata*	2	0.9

*Rhodotorula mucilaginosa*	2	0.9

**Table 5:** Distribution of fungi in HIV positive patients.

Reference number	*Candida* spp.	*Aspergillus* spp.	*Cryptococcus* spp.	*Penicillium* spp.	*Alternaria* spp.	*Rhodotorula* spp.
[9]	69.4%	13.9%	4.2%	—	—	—
[10]	32.5%	57.6%	1.4%	—	—	—
[11]	18.3%	6.9%	0.6%	—	—	—
[12]	55%	3%	4%	—	—	—
[13]	71.7%	14%	1.2%	1.5%	—	0.9%
Our study	86.5%	6.5%	3.3%	1.9%	0.9%	0.9%
